# Decreased exercise capacity in young athletes using self-adapted mouthguards

**DOI:** 10.1007/s00421-021-04659-8

**Published:** 2021-03-13

**Authors:** Johannes Lässing, Roberto Falz, Antina Schulze, Christoph Pökel, Maximilian Vondran, Thomas Schröter, Michael A. Borger, Martin Busse

**Affiliations:** 1 Institute of Sport Medicine and Prevention, University of Leipzig, Marschnerstraße 29a, 04109 Leipzig, Germany; 2 University Department for Cardiac Surgery, Heart Center Leipzig, Leipzig, Germany; 3 Department of Cardiovascular Surgery, Philipps-University Marburg, University Hospital Gießen and Marburg, Marburg, Germany

**Keywords:** Cardiopulmonary compensation, Ventilation, Increased airway resistance, Stroke volume

## Abstract

**Purpose:**

There is evidence of both the preventive effects and poor acceptance of mouthguards. There are various effects on performance depending on the type of mouthguard model. Hemodynamic responses to wearing a mouthguard have not been described. The aim of this study was to investigate the effects of self-adapted mouthguards with breathing channels (SAMG_vent_).

**Methods:**

In this randomized crossover study, 17 healthy, active subjects (age 25.12 ± 2.19 years) underwent body plethysmography and performed two incremental exertion tests wearing a (SAMG_vent_) and not wearing (CON) a mouthguard. Blood lactate, spirometrics, and thoracic impedance were measured during these maximum exercise tests.

**Results:**

The mean values using a SAMG_vent_ revealed significantly greater airway resistance compared to CON (0.53 ± 0.16 kPa·L^−1^ vs. 0.35 ± 0.10 kPa·L^−1^, respectively; *p* = < 0.01). At maximum load, ventilation with SAMGv_ent_ was less than CON (118.4 ± 28.17 L min^−1^ vs. 128.2 ± 32.16 L min^−1^, respectively; *p* = < 0.01). At submaximal loads, blood lactate responses with SAMG_vent_ were higher than CON (8.68 ± 2.20 mmol·L^−1^ vs. 7.89 ± 1.65 mmol·L^−1^, respectively; *p* < 0.01). Maximum performance with a SAMG_vent_ was 265.9 ± 59.9 W, and without a mouthguard was 272.9 ± 60.8 W (*p* < 0.01). Maximum stroke volume was higher using a SAMG_vent_ than without using a mouthguard (138.4 ± 29.9 mL vs. 130.2 ± 21.2 mL, respectively; *p* < 0.01).

**Conclusion:**

Use of a self-adapted mouthguard led to increased metabolic effort and a significant reduction in ventilation parameters. Unchanged oxygen uptake may be the result of cardiopulmonary compensation and increased breathing efforts, which slightly affects performance. These results and the obvious preventive effects of mouthguards support their use in sports.

## Introduction

Mouthguards (MGs) are a key factor in preventing sports-related dental injuries, especially in contact sports (Galic et al. 2018; Lässing et al. 2020b; Petrović et al. 2016). Various studies have demonstrated their preventive effect convincingly (ADA 2006; Bemelmanns and Pfeiffer 2000; Knapik et al. 2007; Lang et al. 2002; Mihalik et al. 2007). However, many athletes are very reluctant to wear mouthguards, largely because of both breathing restrictions (Amis et al. 2000; Bailey et al. 2015; Francis and Brasher 1991) and the fear of impairing performance (Caneppele et al. 2017; Delaney and Montgomery 2019). These limitations seem to depend on the model. There are two main types of mouthguards. Customized mouthguards are worn in professional sports and made individually by dentists. Inexpensive self-adapted mouthguards (SAMG) were designed for self-manufacture and widespread use, especially in youth sports (Kececi et al. 2005; Newsome et al. 2001). Some studies have postulated that using a customized mouthguard (CMG) exerts no negative effects on breathing ($\dot{V}$_E_), oxygen uptake ($\dot{V}$O_2_) or maximum performance compared to wearing a conventional self-adapted mouthguard, or not (Arent et al. 2010; Caneppele et al. 2017; Duarte-Pereira et al. 2008; El-ashke and El-ashker 2015; Morales et al. 2015). In activities involving and requiring high forces or metabolic energy efficiency, even the use of a CMG has demonstrated maximum ergogenic effects (Allen et al. 2018; Buscà et al. 2018; Garner and McDivitt 2009). Described are the hypothetical effects of CMG caused by an increase in airway diameter (Garner and McDivitt 2009) showing positive effects for gas exchange (Garner 2015; Garner et al. 2011; Schulze et al. 2019a), and enhancement through the jaw repositioning associated with beneficial effects on peripheral muscle innervation (Allen et al. 2018; Arent et al. 2010; Morales et al. 2015).

Regarding SAMG use, studies reveal some $\dot{V}$_E_ restriction, but no negative effect on $\dot{V}$O_2_ or performances (Bailey et al. 2015; Francis and Brasher 1991; Schulze et al. 2019a,b,^2020^). In particular, the use of a specially designed SAMG with breathing channels (SAMG_vent_) led to—despite lower $\dot{V}$_E_—a lower blood lactate concentration (Bailey et al. 2015; Schulze et al. 2019a,b,^2020^). Yet other studies have confirmed negative effects on $\dot{V}$O_2_, $\dot{V}$_E,_ and performance from using SAMGs compared to CMG (Bourdin et al. 2006; Caneppele et al. 2017; Duarte-Pereira et al. 2008; Lässing et al. 2020b; Arx et al. 2008).

Hemodynamic parameters associated with the use of mouthguards have not been measured to date. However, documenting these cardiac parameters might give us deeper insight into the effects of self–adapted mouthguard use—effects that might be closely associated with an increase in airway resistance (Bailey et al. 2015; Francis and Brasher 1991). The use of face masks also increases airway resistance, and has shown partially altered hemodynamic parameters (Fikenzer et al. 2020; Lässing et al. 2020a). The aim of this study was therefore to investigate the influence on hemodynamic and metabolic parameters of self-adapted mouthguards with breathing channels (SAMG_vent_). As the effects of wearing mouthguards on pulmonary parameters are known, we would expect a negative impact on performance.

## Materials and methods

### Ethical approval and study group

This study was reviewed and approved by the Ethics Committee of the Medical Faculty at the University of Leipzig (file number 445-15-21122015). All subjects with infectious, orthopedic, intrinsic or other diseases were excluded from this study.

This prospective, randomized, crossover trial investigated the effects of a SAMG_vent_ on cardiopulmonary, metabolic, and maximum power output in an ergometer step test compared to its execution without a mouthguard. The study included 17 healthy subjects (age 25.12 ± 1.9 years, weight 71.82 ± 10.50 kg and height 175.29 ± 8.04 cm). The group consisted of 8 men and 9 women who were sport students and who trained about 3.5 h a week. None of the subjects was a trained cyclist**.** Written informed consent was obtained from all participants. The subjects were advised not to train 24 h before the tests started, and to consume a specific amount of carbohydrates (men 10 g per kg BW and women 7 g per kg BW) to ensure that glycogen conditions remained stable.

### Making of the mouthguards

The self-adapted mouthguard (Nike Adult Max Intake/Beaverton OR, USA) subjects wore is a non-customized mouthguard with breathing channels (SAMG_vent_). They were warmed up in boiling water (30 s) and pressed into the upper jaw by a specialist.

### Body plethysmography

Body plethysmography (ZAN500 Body, nSpire Health GmbH, Germany) measurements were taken with the subject wearing a mask instead of a tube (Lässing et al. 2020c).

**Fig. 1:**
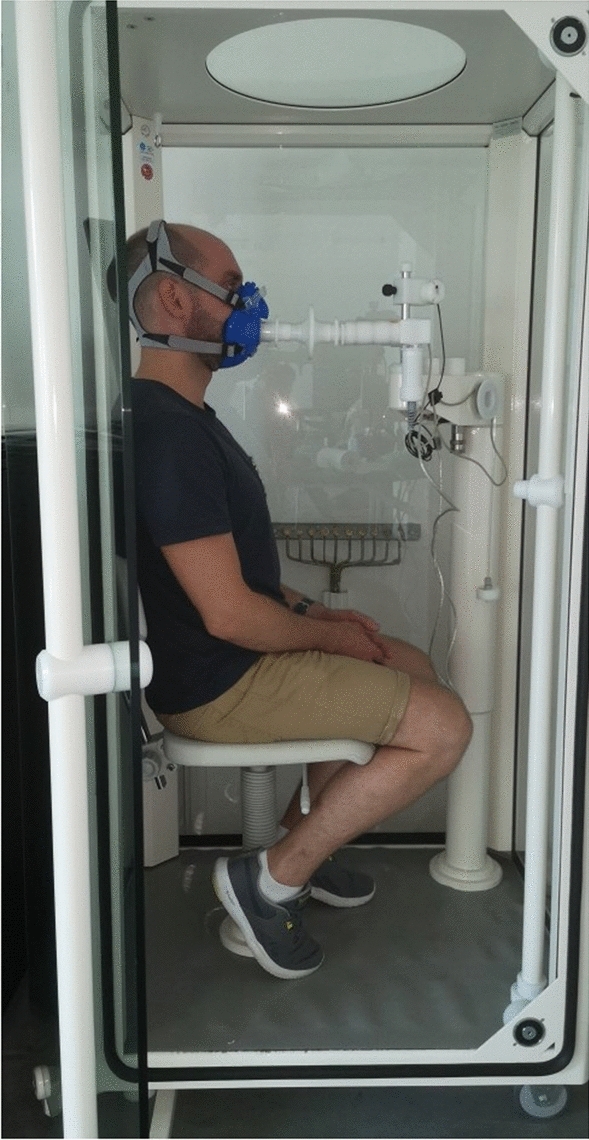
Body plethysmography measurements with spirometry masks

Pulmonary airway resistance (R_AW_) was tested randomly without a mouthguard and with the SAMG_vent_. Between these randomized tests, subjects were given a 5-min break so that their respiratory muscles could recover. The body plethysmography measurements were taken with the participants wearing multi-use silicone face masks with headgear (K4b^2^—face mask, Cosmed, Italy). The test person in Fig. 1 gave his written informed consent allowing his image to appear in an online publication.

### Performance measures

The incremental exercise test was performed on two different days. We allowed an at least 2-day time interval between each test day.

Each test was started with 50 W for men and 30 W for women. Wattage was increased every minute by 15 W for men and 10 W for women up to the maximum possible load. All tests were performed on a semi-recumbent revolution independent cycle ergometer (ergometrics 900, Ergoline GmbH, Bitz, Germany) at 60–70 revolutions per minute. Cardiac output (CO), stroke volume (SV) and heart rate (HR) (measured by impedance cardiography; Physioflow, Manatec Biomedical, Macheren, France), maximum oxygen consumption ($\dot{V}$O_2_ max) and respiratory parameters ($\dot{V}$_E_, *V*_T_, RR) were monitored continuously at rest and during stress (K4b^2^, Cosmed, Italy). Spirometric and thoracic impedance data were averaged for 10 s over the load.

To monitor cardiac arrhythmias, the C5-lead ECG was continuously observed to ensure the subjects’ preventive forensic safety. Blood-lactate samples (20 µL) were taken every three minutes and subjected to enzymatic-amperometric measurement (Super GL, ISO 7550, Germany). Blood pressure (BP) was measured under rest, every three minutes under stress, and after the workload. Load intensity was classified as: “rest” (0 W), “moderate” (men = 215 W/women 170 W), “submaximal” (men MW = 320 W/women MW = 210 W) and individual “maximum”.

### Calculations

Spirometric and thoracic impedance data were recorded as the 1-min average for each load level.

We calculated alveolar ventilation ($\dot{V}$_A_) by relying on the spirometrically recorded parameters that applied in these calculations (Bohr-formula): dead space volume (VD = *V*_T_ × [FetCO_2_ (end-tidal fractional carbon dioxide concentration) − FeCO_2_ (mixed expired carbon dioxide concentration]/FetCO_2_), dead space ventilation ($\dot{V}$D = VD × RF); alveolar ventilation ($\dot{V}$_A_ = (V_T_ − VD) × RF). Breathing effort was calculated as follows: Intrapulmonary pressure = PEF × RAW. TPR was calculated: TPR = MAP/CO.

### Statistical analysis

All values are presented as means with standard deviation. GraphPad Prism 8 (GraphPad Software Inc., California, USA) was used for statistical evaluations and graph preparation. The raw data from spirometry and impedance cardiography obtained continuously during exercise were synchronized and averaged over 10 s. The exercise parameters were then calculated for all subjects at moderate, submaximal and maximum load. For distribution analysis, the Kolmogorov–Smirnov normality test was used. If normality distribution was evident, statistical comparisons were made using paired parametric *t* test (body plethysmography, significance level was defined as *p* < 0.05) or repeated two-way ANOVA with Bonferroni´s post hoc test for multiple comparison (exercise parameter). Sphericity was determined-based on the epsilon value of the Geisser greenhouse (*ε*). If the sphericity was rejected, Greenhouse Geisser correction would apply.

## Results

### Body plethysmography measured with mask

Table 1 illustrates pulmonary parameters in the body plethysmography measurement with mask. Pulmonary function parameters showed no differences. Only airway resistance was significantly higher with the SAMG (Table 1).

**Table 1:** Body plethysmography measurement using the mask

Body plethysmography	CON	SAMG_vent_	${\eta }_{\mathrm{p}}^{2}$	*p* value
R_AW_ (kPa·L^−1^)	0.35 ± 0.10	0.53 ± 0.16	0.57	** < 0.01**
VC (L)	5.06 ± 1.03	4.89 ± 0.95	0.17	0.09
FEV_1_ (L)	3.91 ± 0.65	3.82 ± 0.73	0.09	0.23
FEV_1_/FVC	79.71 ± 4.74	80.65 ± 4.76	0.08	0.26
P_EF_ (L·s^−1^)	8.19 ± 1.54	8.27 ± 2.23	< 0.01	0.78
P_IF_ (L·s^−1^)	4.39 ± 1.73	4.06 ± 1.84	0.04	0.49

Values are presented as the means and standard deviation

Significant difference in bold, SAMG_*vent*_ self-adapted mouthguard with breathing channels, *CON* without mouthguard, *SD* standard deviation, *VC* vital capacity, *R*_*AW*_ airway resistance, *FEV*_*1*_ forced expiratory volume in one second, *FVC* forced vital capacity, *FEV*_*1*_*/FVC* Tiffeneau–Pinelli index, *P*_*EF*_ peak flow (expiratory), *P*_*IF*_ peak flow (inspiratory), *η*^*2*^_*p*_ partial eta squared

Respiratory work was calculated relying on peak flow and airway resistance parameters, which revealed significant differences (CON 2.78 ± 0.8 kPa vs. SAMG_vent_ 4.27 ± 1.7 kPa, *p* = < 0.01/*η*_p_^2^ = 0.48).

### Exercise testing

**Fig. 2:**
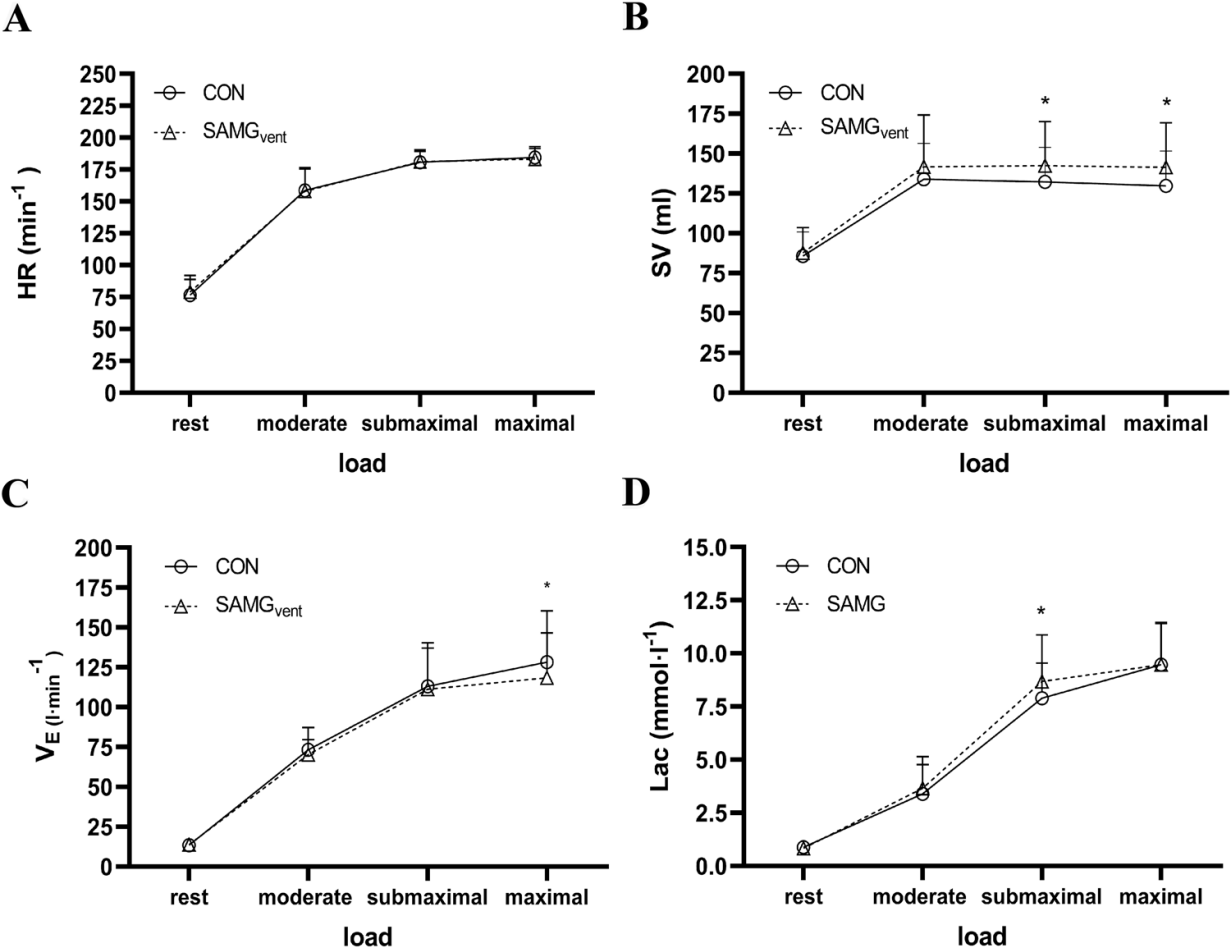
Tow-way ANOVA with mean values and standard deviation: **a** HR during rest and stress, **b** stroke volume during rest and stress, **c** ventilation during rest and stress, **d** lactate during rest and stress. Asterisk significant differences at the respective level

Baseline values were measured prior to each session (values not shown), and there were only TPR and *T*_e_ significant differences in hemodynamics. 17 participants completed both tests. Figure 2 shows the time course of HR, SV, *V*_E_ and Lac during the exercise tests with and without mouthgard. There were no significant differences in hemodynamics or metabolic parameters during moderate intensity. TPR was significantly lower at rest with SAMG_vent_ (CON 15.62 ± 3.55 mmHg·L^−1^ vs. SAMG_vent_ 14.15 ± 2.59 mmHg·L^−1^). *T*_e_ was clearly prolonged under resting conditions with mouthguard use (CON 2.23 ± 1.13 s vs. SAMG_vent_ 2.37 ± 1.12 s; *p* = 0.04). At submaximal intensity, LAC (CON 9.89 ± 1.65 mmol L^−1^ vs. SAMG_vent_ 8.68 ± 2.20 mmol L^−1^; *p* < 0.01) and SV (CON 132.1 ± 20.9 mL vs. SAMG_vent_ 139.4 ± 29.7 mL; *p* = 0.02) showed differences. All other measured parameters were at submaximal intensity not statistically different. Systolic and diastolic blood pressure revealed no differences throughout the exercise tests. Table 2 shows the maximum exercise parameters. The maximum power output achieved was lower with an SAMG. Pulmonary parameters differed significantly except for VO_2_ and *V*_T_. The SV was significantly increased and AVDO_2_ decreased when wearing an SAMG_vent_.

**Table 2:** Exercise results with and without a self–adapted mouthguard

	CON	SAMG_vent_		Adjusted *p* value
Pulmonary parameters				
$\dot{V}$O_2_ (mL min^−1^ kg^−1^)	48.27 ± 7.13	48.28 ± 7.91		> 0.99
FETO_2_ (%)	16.28 ± 0.58	16.02 ± 0.72		**0.04**
FETCO_2_ (%)	5.07 ± 0.59	5.33 ± 0.63		** < 0.01**
$\dot{V}$_E_ (L·min^−1^)	128.2 ± 32.16	118.4 ± 28.17		** < 0.01**
RF (bpm)	48.65 ± 9.23	44.29 ± 6.39		** < 0.01**
*V*_T_ (L)	2.66 ± 0.45	2.69 ± 0.58		> 0.99
*T*_i_ (s)	0.62 ± 0.10	0.67 ± 0.09		**0.03**
$\dot{V}$A (L·min^−1^)	96.90 ± 24.3	85.82 ± 20.6		** < 0.01**
*T*_e_ (s)	0.68 ± 0.13	0.74 ± 0.12		> 0.99
Hemodynamics parameters				
HR (min^−1^)	184.5 ± 8.6	183.2 ± 8.3		0.70
CO (L·min^−1^)	24.0 ± 3.6	25.2 ± 5.1		0.10
SV (mL), *n* = 16	129.8 ± 21.8	141.4 ± 28.0		** < 0.01**
LAC (mmol·L^−1^)	9.48 ± 1.93	9.47 ± 1.99		> 0.99
AVDO_2_ (%)	14.70 ± 3.09	14.03 ± 3.05		** < 0.01**
TPR mmHg·L^−1^	5.30 ± 0.84	5.12 ± 1.17		> 0.99
Peak power output (W)	272.9 ± 60.8	265.9 ± 59.9		**0.01**

Values presented as the means and standard deviation; adjusted *p* value = ANOVA with Bonferroni’s post hoc, moderate and submaximal values not shown

Significant difference in bold, SAMG_*vent*_ self-adapted mouthguards with breathing channels, *CON* without mouthguard, *mean* group mean values, *SD* standard deviation, $\dot{V}$*O*_*2*_ oxygen uptake/min, *RF* respiratory frequency, *V*_*T*_ tidal volume, $\dot{V}$_*E*_ ventilation/min, *T*_*i*_ inspiratory time, *T*_*e*_ expiratory time, *FetO*_*2*_ end-tidal fractional oxygen concentration, *FetCO*_*2*_ end-tidal fractional carbon dioxide concentration, *HR* heart rate, *RQ* respiratory quotient, *SV* stroke volume, *CO* cardiac output, *AVDO*_*2*_ arteriovenous oxygen difference, *Lac* blood lactate concentration, *SBP* systolic blood pressure, *DBP* diastolic blood pressure, $\dot{V}$*A* alveolar ventilation, *TPR* total peripheral resistance, *CW* cardiac work

## Discussion

Our study’s main finding was that wearing a self-adapted mouthguard significantly increases airway resistance (R_AW_) at rest and reduces the $\dot{V}$_E_ during maximum load. Despite similar $\dot{V}$O_2_ values, we observed a small but significantly reduced maximum ergometer performance when the SAMG_vent_ was worn. Cardiopulmonary and metabolic parameters (Fig. 2) may indicate primarily mechanical and less peripheral neural autonomic compensation to maintain $\dot{V}$O_2_ when wearing an SAMG_vent_.

### Pulmonary parameter

Body plethysmography revealed that *R*_AW_ rises significantly when wearing an SAMG_vent_. Other studies have also reported a significant or trending increase with MGs (Amis et al. 2000; Lässing et al. 2020c). Respiratory protection devices and breathing filters reveal similar effects (Lee and Wang 2011; Louhevaara 1984). Those studies demonstrate that increased *R*_AW_ can also significantly reduce $\dot{V}$_E_ during exercise, and lower the athlete’s performance (Fikenzer et al. 2020; Lässing et al. 2020c; Louhevaara 1984; Melissant et al. 1998). Such significantly lower $\dot{V}$_E_ confirms the present study’s findings when using an SAMG (Bailey et al. 2015; Caneppele et al. 2017; Delaney and Montgomery 2019; Francis and Brasher 1991; Schulze et al. 2020). RF was also clearly reduced in conjunction with SAMG_vent_ use, whereas *V*_T_ was not influenced at maximum workload. Note that other studies have also reported lower RF with corresponding changes in breathing time when face-protection devices were used (Amis et al. 2000; Fikenzer et al. 2020; Francis and Brasher 1991; Lässing et al. 2020c; Louhevaara 1984; Schulze et al. 2020). Francis and Brasher (1991) suggest that a prolonged breathing cycle is a compensatory mechanism that can stabilize *V*_T_ and the gas exchange when wearing an SAMG (Amis et al. 2000; Bailey et al. 2015; Lässing et al. 2020c). There is also evidence that CMG use had no effects on *V*_T_ and $\dot{V}$O_2_ under maximum ergometer performance, but it did reduce $\dot{V}$_E_ and extend *T*_i_ (Lässing et al. 2020c). According to Francis and Brasher (1991), a mechanism resembling the 'pursed lip' type of breathing (PLB) in patients with obstructed breathing lengthens the respiratory cycle time. The present results demonstrate reduced $\dot{V}$_A_, $\dot{V}$_E_, prolonged *T*_i_, and lower performance with a SAMG_vent_ compared to CON despite similar $\dot{V}$O_2_. The most likely explanation for these changes is the significantly increased airway resistance. Even more, the resulting greater breathing effort needed to maintain VE cancels some cardiopulmonal capacity, and might lead to distributional congruence between the respiratory and peripheral muscles (reduced AVDO_2_ and increased lactate) (Dominelli et al. 2017). The reduced ergometer performance despite unchanged *V*O_2_ may be attributable to this.

In the present study, the FetO_2_ was lower and FetCO_2_ clearly increased with the SAMG_vent_, compared to without a mouthguard. Some researchers have reported similar results, and assume an improved gas exchange rate when wearing a mouthguard (Garner et al. 2011; Schulze et al. 2020). Schulze et al. (2020) suspect that an altered jaw position favors innervation in the temporomandibular joint and associated dorsal muscle chain. They hypothesize that improved peripheral control stimulates the aerobic metabolic pathway, which may explain higher CO_2_ production per breath (Schulze et al. 2020). The present results indicate minor but significantly higher lactate levels, as well as 2.6% less maximum power output using an SAMG_vent_. The obstructive breathing patterns may be the reason for higher alveolar carbon dioxide partial pressure, represented by the FETCO_2_ value.

By wearing an SAMG_vent_ higher *R*_AW_ values lead to an altered exercise breathing pattern and significantly increased breathing capacity in healthy subjects, which limits $\dot{V}$_A_ but not $\dot{V}$O_2_ (Bailey et al. 2015; Francis and Brasher 1991; Lässing et al. 2020c; Schulze et al. 2020).

### Cardiocirculatory and metabolic parameters

There were no differences in HR parameters associated with wearing a mouthguard (Bailey et al. 2015; Delaney and Montgomery 2019; El-ashke and El-ashker 2015; Lässing et al. 2020c) in this study. Others have speculated that the PLB mechanism may influence performance when a mouthguard is worn (Amis et al. 2000; Bailey et al. 2015; Delaney and Montgomery 2019; Francis and Brasher 1991). We observed a higher SV in conjunction with SAMG_vent_ use. Respiration is known to affect the SV (Convertino et al. 2005; Fikenzer et al. 2020; Jayaweera and Ehrlich 1987; Lässing et al. 2020c; Ryan et al. 2008). Some authors suspect that a longer *T*_i_ keeps pleural pressure on a negative level for longer, and may thus favor venous return (Jayaweera and Ehrlich 1987) during mouthguard use (Lässing et al. 2020c). Other studies have shown that increased inspiratory airway resistance can raise the SV (Convertino et al. 2005; Ryan et al. 2008). Increased respiratory muscle effort because of neural-reflex mechanisms could also be responsible for the rise in SV (Harms et al. 1998; Lee and Wang 2011). Unchanged blood pressure values and similar HRs suggest a more cardiopulmonary-mechanical than neural-reflex mechanism (Ryan et al. 2008). TPR’s mean values did not differ during exertion, thus supporting the assumption of a mechanical factor rather than a neuronal effect. As respiratory resistance induced a prolonged inspiratory phase, this could presumably increase the venous return flow and thus explain the mechanically-induced higher SV with enhancing effects on the $\dot{V}$O_2_ and maybe even the performance (Lässing et al. 2020c). The reduced AVDO_2_ during exercise is consistent with other studies reporting increased airway resistance when wearing face masks (Fikenzer et al. 2020; Lässing et al. 2020a). Reduced oxygen extraction caused by ventilatory obstruction has been suggested to be behind the increased lactate levels, and higher CO may due to afferent innervation from the working muscles (Blain et al. 2005; Busse et al. 1991; Harms et al. 1998). In contrast, independent studies demonstrated also the mechanical relationship between longer or higher negative pleural pressure and possible forcing effects on the transmural pressure difference in the extrathoracic and intrathoracic vessels (Convertino et al. 2005; Ryan et al. 2008) which may increase venous blood return and improve SV (Convertino et al. 2005; Fagoni et al. 2020; Fikenzer et al. 2020; Lässing et al. 2020a; Ryan et al. 2008).

In summary: the wearing of an SAMG_vent_ led to an obstructed breathing pattern (Amis et al. 2000; Bailey et al. 2015; Francis and Brasher 1991; Lässing et al. 2020c) indicating slightly reduced maximum power (Caneppele et al. 2017; Duarte-Pereira et al. 2008; El-ashke and El-ashker 2015) without restricting $\dot{V}$O_2_ (Bailey et al. 2015; Francis and Brasher 1991; Kececi et al. 2005; Schulze et al. 2019a,^2020^). Mechanical cardiopulmonary compensation may contribute to stabilizing the $\dot{V}$O_2_ (Convertino et al. 2005; Lässing et al. 2020c; Ryan et al. 2008) which is probably higher because of the increased breathing effort while wearing a mouthguard than with no mouthguard. Nevertheless, the performance of participants wearing an SAMG_vent_ in this study revealed moderate restrictions, probably because of the respiratory muscles’ higher oxygen consumption. As a similar study (Lässing et al. 2020c) employing customized mouthguards (CMG) reported no reduction in performance, we conclude that CMGs are preferable to the SAMG_vent_ in this study.

### Study limitations

The cardiac parameters we obtained via impedance cardiography may have been overestimated using absolute values (Siebenmann et al. 2015). However, since we compared intra-individual differences and impedance cardiography is so reliable (Astorino et al. 2015; Richard et al. 2001), changes in these parameters were essential, unlike those achieved using absolute values. Since to enable separate gender-specific data we would have needed a much larger cohort of study subjects, we cannot evaluate gender-specific differences. Nevertheless, our analyses show large homogeneity in the variation in variance of all means. Furthermore, our work does not take into account long-term adaptive regulations using a mouthguard, since the subjects wore the mouthguard only for these examinations.

## Conclusion

Our investigation revealed increased airway resistance under resting conditions and significantly reduced respiratory parameters under stress in conjunction with wearing an SAMG_vent_. Maximum power output dropped slightly also, while the blood lactate concentration was higher. Oxygen uptake was unchanged and stroke volume improved, factors that potentially indicate cardiopulmonary compensation in combination with increased breathing effort. Nevertheless, we have demonstrated that wearing an SAMG_vent_ reduces performance moderately—a factor that should be considered when these models are being used in sports.

## Abbreviations

ADA: Access, prevention and interprofessional relations
AVDO_2_: Arteriovenous oxygen difference
CAP: Concurrent activation potentiation
CO: Cardiac output
FetCO_2_: End-tidal fractional carbon dioxide concentration
FetO_2_: End-tidal fractional oxygen concentration
FEV_1_: Forced expiratory volume in one second
FVC: Forced vital capacity
HR: Heart rate
SAMG_vent_: Self-adapted mouthguard with breathing channels
CON: Control (without mouthguard)
P_EF_: Peak expiratory flow
P_IF_: Peak inspiratory flow
R_AW_: Airway resistance
RF: Respiratory frequency
RQ: Respiratory quotient
SD: Standard deviation
SV: Stroke volume
*T*_e_: Expiratory time
*T*_i_: Inspiratory time
TPR: Total peripheral resistance
$$\dot{V}$$_A_: Alveolar ventilation
VC: Vital capacity
$\dot{V}$CO_2_: Carbon dioxide production
$$\dot{V}$$_E_: Ventilation
$$\dot{V}$$O_2_: Oxygen uptake
$\dot{V}$O_2max_: Maximum oxygen uptake
*V*_T_: Tidal volume
W: Watt

## Declarations

### Availability of data and material

The datasets used and/or analyzed during the current study are available from the corresponding author upon reasonable request.
